# Tryptamine Attenuates Experimental Multiple Sclerosis Through Activation of Aryl Hydrocarbon Receptor

**DOI:** 10.3389/fphar.2020.619265

**Published:** 2021-01-25

**Authors:** Nicholas Dopkins, William Becker, Kathryn Miranda, Mike Walla, Prakash Nagarkatti, Mitzi Nagarkatti

**Affiliations:** ^1^ Department of Pathology, Microbiology and Immunology, University of South Carolina School of Medicine, Columbia, SC, United States; ^2^ Department of Chemistry and Biochemistry, University of South Carolina, Columbia, SC, United States

**Keywords:** neuroinflammation, tryptamine, experimental autoimmune encephalitis, aryl hydrocarbon receptor, autoimmunity

## Abstract

Tryptamine is a naturally occurring monoamine alkaloid which has been shown to act as an aryl hydrocarbon receptor (AHR) agonist. It is produced in large quantities from the catabolism of the essential amino acid tryptophan by commensal microorganisms within the gastrointestinal (GI) tract of homeothermic organisms. Previous studies have established microbiota derived AHR ligands as potent regulators of neuroinflammation, further defining the role the gut-brain axis plays in the complex etiology in multiple sclerosis (MS) progression. In the current study, we tested the ability of tryptamine to ameliorate symptoms of experimental autoimmune encephalomyelitis (EAE), a murine model of MS. We found that tryptamine administration attenuated clinical signs of paralysis in EAE mice, decreased the number of infiltrating CD4^+^ T cells in the CNS, Th17 cells, and RORγ T cells while increasing FoxP3+Tregs. To test if tryptamine acts through AHR, myelin oligodendrocyte glycoprotein (MOG)-sensitized T cells from wild-type or Lck-Cre AHR^flox/flox^ mice that lacked AHR expression in T cells, and cultured with tryptamine, were transferred into wild-type mice to induce passive EAE. It was noted that in these experiments, while cells from wild-type mice treated with tryptamine caused marked decrease in paralysis and attenuated neuroinflammation in passive EAE, similar cells from Lck-Cre AHR^flox/flox^ mice treated with tryptamine, induced significant paralysis symptoms and heightened neuroinflammation. Tryptamine treatment also caused alterations in the gut microbiota and promoted butyrate production. Together, the current study demonstrates for the first time that tryptamine administration attenuates EAE by activating AHR and suppressing neuroinflammation.

## Introduction

MS is an incurable autoimmune disorder in which the immune system recognizes the myelin sheath surrounding neurons, instigating an inadvertent cascade of pathogenic inflammation within the central nervous system (CNS). Inflammatory cascades in advanced MS accumulate to form plaques and lesions composed of demyelinated tissue in the CNS that hinder nervous tissue function. Symptoms arising from an advanced MS state include general discomfort, wasting, cognitive decline and paralysis due to hindered ability to produce action potentials in the CNS regions that innervate homeostatic, intellectual and sensory processes (Reich et al., 2018). The aforementioned inflammatory cascade is characterized by a T lymphocyte landscape defined primarily by a disproportionate abundance of inflammatory T Helper 17 (Th17) cells infiltrating the CNS and further perpetuating the pro-inflammatory adaptive immune response (Dos Passos et al., 2016). Antiparallel to the overabundance of the Th17 cells driving neuroinflammation is an absence of activity by Tregs that suppress inflammation, and confer neuroprotection by the direct inhibition of adaptive immunity (Costantino et al., 2008; Li et al., 2019).

While the precise mechanisms of etiology of MS and autoimmune disorders is unclear, there is a growing body of evidence which suggests that environmental factors contribute significantly to the outcome and onset of autoimmune disease states (O’Gorman et al., 2012; Correale et al., 2017). One of these external factors that plays a critical role in regulating autoimmunity is the microorganisms residing within the GI tract referred to as the “gut microbiota”. The gut microbiota has been established by previous studies to be an important external factor that influences the Treg/Th17 balance and regulates T cell immunity within the context of autoimmune disorders (Pandiyan et al., 2019). Advances in sequencing technologies, mass spectrometry and germ-free facilities have expedited the ability of researchers to better define the potential of the microbiota in limiting excessive inflammation in autoimmune disorders, including MS (Ochoa-Repáraz et al., 2010; Castillo-Álvarez and Marzo-Sola, 2017; Dopkins et al., 2018; Kirby and Ochoa-Repáraz, 2018; Dehner et al., 2019). In particular, previous studies have demonstrated that a healthy microbiota limits excessive inflammation and alleviates inflammatory disorder pathology by inducing Tregs via the excretion of short chain fatty acids (SCFAs) and immunomodulatory AHR ligands (Vinolo et al., 2011; Rothhammer and Quintana, 2016). However, the potential of the microbiome in inflammatory disorders serves as a double-edged sword due to chronic intestinal dysbiosis being demonstrated to worsen and instigate inflammatory disorders (Lee et al., 2011; Li et al., 2018; Opazo et al., 2018; Saltzman et al., 2018; André et al., 2019). These previous studies have established that disparity in the microbial composition along barrier sites is directly involved with the clinical outcome of inflammatory disorders afflicting the host organism, and therefore suggest further studies focused on the etiological role of the microbiome in inflammatory diseases in order to develop novel therapeutics and identify biomarkers for early detection.

For this purpose, in the current study, we investigated the anti-inflammatory potential of an AHR agonist produced by the gut microbiota, tryptamine, in the limiting of autoimmune neuroinflammation in experimental MS. There is little known about the effects of tryptamine on host immunity and GI tract function despite tryptamine being produced in large quantities by the gut microbiota of >10% of the population (Jin et al., 2014; Williams et al., 2014; Gao et al., 2018). In this study, we found that tryptamine suppresses neuroinflammation and attenuates EAE by acting through AHR. To the authors knowledge this is the first report demonstrating any *in vivo* immunosuppressive properties of tryptamine in a state of acute or chronic autoimmunity.

## Materials and Methods

### Mice

Six- to eight-week-old female C57BL/6 mice were purchased from Jackson Laboratories (Bar Harbor, ME) and housed within the Animal Resource Facilities (ARF) at the University of South Carolina School of Medicine. Mice were cohoused for a period of 2 weeks prior to experimentation. On day 0, mice were randomly divided into either vehicle or treatment groups prior to the induction of EAE. Lck-Cre AHR^flox/flox^ mice on C57Bl/6 background were bred in house using AHR^tm3.1Bra^/J (Jackson stock number 006203) and dLCK-hcre^3779^/J (Jackson stock number 012837) mice purchased from Jackson Laboratories. The resulting Lck-Cre AHR^flox/flox^ are a cell-specific knockout that lack expression of a functional AHR in all T Cells. All donor Lck-Cre AHR^flox/flox^ were age matched with WT donor mice prior to immunization for all passive EAE experiments. All animal work was conducted in accordance with protocols that follow the National Institute of Health guidelines and were approved by the Institutional Animal Care and Use Committee (IACUC) of the University of South Carolina.

### Reagents

The following reagents were used during the course of the experiments and were purchased as following: red blood cell (RBC) lysis buffer was purchased from Millipore Sigma (Burlington, MA); Percoll from GE Healthcare Life Sciences (Pittsburgh, PA); Myelin oligodendrocyte glycoprotein peptide subunit 35–55 (MOG35-55) from PolyPeptide Laboratories (San Diego, CA); Pertussis Toxin (PTX) from List Biological Laboratories (Campbell, CA); Heat killed *Mycobacterium tuberculosis* (H37Ra) from Difco (Detroit, MI); β-mercaptoethanol, Freund’s Advjuvant, propionic acid, n-butyric acid, isovaleric acid, 2-ethylbutyric acid, sulfuric acid and Tween-80 from Sigma-Aldrich (St. Louis, MO); absolute ethanol, acetic acid, lithium carbonate and tryptamine were purchased from Fisher Scientific (Hamptom, NH); RPMI 1640, fetal bovine serum (FBS), L- glutamine, phosphate buffered saline (PBS), luxol fast blue (LFB), cresyl violet, eosin, hematoxylin, and HEPES were purchased from VWR (West Chester, PA; flourophore conjugated antibodies and ELISA kits from Biolegend (San Diego, CA); Illumina MiSeq reagents from Illumina Inc. (San Diego, CA); QIAamp Stool Mini Kits from Qiagen (Germantown, MD); 10% formalin from Azer Scientific (Morgantown, PA).

### Induction of Chronic Progressive EAE and Treatment With Tryptamine

Chronic progressive EAE was induced in C57BL/6 mice according to previously published protocols (Constantinescu et al., 2011; Glatigny and Bettelli, 2018). Briefly, mice were immunized on day 0 with subcutaneous injections containing 150 µg of myelin oligodendrocyte glycoprotein subunit 35–55 (MOG35-55) and 600 mg heat killed *Mycobacterium tuberculosis* (H37Ra) suspended within an emulsion of PBS and Freund’s complete adjuvant On day 0 and 2, mice received a single intraperitoneal injection containing 200 and 400 ng of PTX, respectively. Beginning on day 1, mice received a 50 µL intraperitoneal injection containing either a vehicle (sterile corn oil (CO) with 2% DMSO v/v) or a treatment suspension (12.5 mg/kg tryptamine in sterile CO with 2% DMSO v/v) every 48 h. This dose of 12.5 mg/kg was chosen in accordance with previous studies that have demonstrated it to be a safe dose that will not induce short term behavioral changes in mice (Mousseau, 1993). This dose was also deemed to be safe and clinically relevant when converted to a human equivalent dose of 1 mg/kg (Nair and Jacob, 2016; Wüst et al., 2017).

### EAE Scoring of Paralysis Symptoms

The weight and paralysis symptoms were recorded within EAE mice daily. The scoring of paralysis symptoms was done according to the following key: 0 = no symptoms, 1 = inability to curl the distal end of tail, 2 = complete tail atony/impaired movement, 3 = partial hind limb paralysis, 4 = complete hind limb paralysis, 5 = tetraplegia/moribund state.

### Cell Isolation

Single cell suspensions were prepared using neural tissue dissociation kits purchased from Miltenyi according to manufacturer instructions (Pennartz et al., 2009). After dissociation of CNS tissue, myelin was removed from the single cell suspensions using two washes in 30% percoll. Remaining cells were then subjected to RBC lysis buffer, washed and suspended in RPMI supplemented with FBS and penicillin/streptomycin (cRPMI) for downstream use. Immune cells were isolated from the spleens and lymph nodes by use of mechanical dissociation followed by RBC lysis and filtration using 70 µm filters. After washing, the remaining mononuclear fractions were suspended in cRPMI for downstream use.

### ELISA Quantification of Secreted Cytokines

Media was collected from primary cultures of mononuclear cells isolated from the CNS and spleen plated at 10 × 10^6^ cells/ml under unstimulated conditions from EAE mice treated *in vivo* and MOG35-55 (20 µg/ml) stimulated conditions from MOG35-55 immunized mice treated *in vitro* for ELISA quantification of IL-10 and IL-17. ELISAs were performed according to Biolegend protocols. Briefly, high affinity protein-binding plates were coated by incubating a 100 µL suspension of capture antibody overnight at 4°C. The plates were then washed three times using wash solution of (PBS + 0.05% Tween80). The plates were then incubated with blocking solution for 1 h at room temperature (RT). After incubation, the plate was washed three times using wash solution and incubated for 2 h at RT with 100 µL of standards prepared using serial dilutions according to manufacturer instructions and 100 µL of supernatant collected from overnight cell cultures. Plates were again washed three times with wash solution before incubating with 100 µL of a biotinylated detection antibody solution diluted according to the manufacture’s instruction in blocking solution at RT for 1 h. After incubation with the detection antibody, plates were washed three times with solution and incubated with horseradish peroxidase (HRP) conjugated avidin antibody for 30 min at RT. After incubation with HRP-avidin, the ELISA plates were washed five times with wash solution and incubated with TMB substrate for fast color development. After color development, the reaction was stopped simultaneously in all wells using 1N hydrosulfuric acid. The concentration of captured protein content was calculated by comparing relative absorbance of variable samples at a wavelength of 450 nm to the standard curve calculated from standards of known concentration. Plates were analyzed with a PerkinElmer Victor^2^ plate reader.

### Flow Cytometry for Quantification of Immune Cell Phenotypes

Suspensions of single mononuclear cells isolated from splenic and CNS tissue using previously described methods were phenotyped using a BD FACSCelesta flow cytometer. Single cell suspensions were tagged with fluorescently labeled monoclonal antibodies purchases from Biolegend (APC conjugated anti-RORγT, BV786 conjugated anti-CD4, FITC conjugated anti-CD3, BV421 conjugated anti-Foxp3, PE conjugated and BV605 conjugated anti-CD45). Analysis of .fcs files was conducted using FlowJo software.

### Stimulation of MOG35-55 Reactive Mononuclear Cells *In Vitro*

C57BL/6 mice were immunized with subcutaneous injections of 150 µg of MOG35-55 and 600 mg H37Ra suspended within an emulsion of sterile PBS and Freund’s adjuvant. After 7 days, the mice were euthanized using isoflurane. Spleens were excised from the mice and cells were isolated via mechanical dissociation, RBC lysis and 70 µm filtration. Live cells were plated at 10 × 10^6^ cells/ml in complete cRPMI activated with 20 µg/ml MOG35-55. Cells were activated in the presence or absence of 100 µM tryptamine in the medium and cultured for 48 h. Statistical analysis was performed using paired *t*-tests to compare changes across donor samples in the presence or absence of tryptamine.

### Induction of Passive Polyclonal EAE

Passive polyclonal EAE was induced as previously described (McPherson et al., 2014). Briefly, C57BL/6 (wild-type) and Lck-Cre AHR^flox/flox^ mice were immunized with subcutaneous injections of 150 µg of MOG35-55 and 600 mg H37Ra suspended within an emulsion of sterile PBS and Freund’s complete adjuvant. After 7 days, the mice were humanely euthanized. Cells were isolated from the spleens and inguinal lymph nodes of these donor mice as previously described. Live cells were incubated at a concentration of 4 × 10^6^ cells/ml in cRPMI medium supplemented with MOG35-55 (10 µg/ml), rIL-2 (10 U/ml), rIL-12 (25 ng/ml) and rIL-18 (25 ng/ml) in the presence or absence of tryptamine (100 µM). After 48 h, the cells were washed and plated in cRPMI medium supplemented with MOG35–55 (10 µg/ml), rIL-2 (20 U/ml), rIL-12 (25 ng/ml) and rIL-18 (25 ng/ml) in the presence or absence of tryptamine (100 µM). After 24 h, the cells were washed, counted and brought to a final concentration of 4 × 10^6^ live cells/100 µL within sterile PBS. 4 × 10^6^ live cells were given via retro-orbital injection to recipient C57BL/6 (wild-type) mice. After 2 h, mice received an intraperitoneal injection containing 200 ng of PTX suspended within sterile PBS. After 48 h, the mice received a second intraperitoneal injection containing 400 ng of PTX suspended in sterile PBS. Paralysis symptoms were recorded daily as previously described.

### Histological Analysis

Euthanized mice were perfused with 10 ml of heparinized PBS followed by 10 ml of 10% formalin. After isolation, whole brains were held in 70% EtOH and embedded in paraffin blocks. 7 µm sections were cut using a microtome and placed onto microscope slides using a water bath. Slides were deparaffinized with washes of xylene and ethanol prior to staining with Luxol Fast Blue and Hematoxylin and Eosin (H&E) according to previously published protocols (Feldman and Wolfe, 2014; Carriel et al., 2017).

### Bacterial Phylogenetic Profiling by 16s Analysis

Phylogenetic profiling of the cecal microbiota was conducted by use of 16s rDNA sequencing of DNA isolated from cecal contents of vehicle and tryptamine treated mice. Briefly, DNA was isolated from cecal contents using the QIAamp Stool Mini Kit according to the manufacturer instructions. Sequencing was performed using the Illumina MiSeq platform. Downstream analysis of .fastq files was conducted using the Nephele platform provided via the National Institute of Allergy and Infectious Diseases (NIAID) (Weber et al., 2018). Further downstream analysis of Nephele operational taxonomic unit (OTU) outputs was conducted using linear discrimination of effect size (LEfSe) analysis (Segata et al., 2011).

### Quantification of SCFAs

SCFA content was quantified from cecal content using methods previously described (Zhao et al., 2006; Busbee et al., 2020). Briefly, 100 mg of cecal content was homogenized in 400 µL deionized H20. After homogenization, samples were acidified by adding 1:4 of the total volume of 25% metaphosphoric acid. After 30 min of incubation, samples were centrifuged (12,000 g for 15 min), with the supernatant being collected and filtered using Ultra-free MC Columns (ThermoFisher). Ethyl butyric acid was added to each sample at a final concentration of 0.0375 mM for use as an internal standard to accurately calculate experimental concentrations. Samples then received 400 µL of methyl tert-butyl ether (MTBE) and were vortexed vigorously. Samples were then spun at 400 g for 5 min. Lastly, 100 µL of the upper organic layer was transferred to a fresh glass tube. Samples were then analyzed with an HP 5890 gas chromatograph configured with flame-ionized detection (GC-FID).

### Statistical Analysis

Statistical analysis was conducted using GraphPad Prism software version 8.4.3. Unpaired t tests were used to compare experimental groups separated by a single variable. Paired t tests were used to compare changes in experimental groups that shared a single subject prior to subjection to a variable. Common subjects are annotated by a solid line connecting individual values across groups. To test the efficacy of tryptamine in EAE, we used four experiments containing five mice per group equating to a total of 20 mice. The number of animals used in other experiments have been shown in Figure legends. All graphs show individual values with the mean ± standard error of the mean (SEM). Samples were considered statistically significant if *p* < 0.05. Degree of significance was demonstrated using the following key: *p < 0.05, **p < 0.01, ***p < 0.001.

## Results

### Tryptamine Ameliorates EAE Severity

**FIGURE 1:**
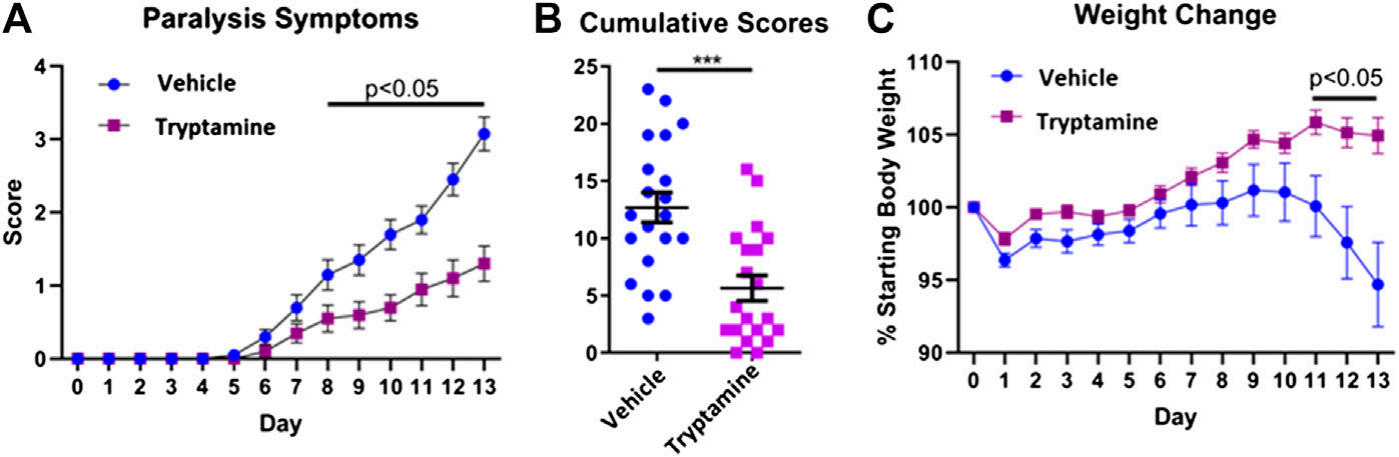
Tryptamine ameliorates EAE severity. **(A)** Paralysis symptoms of vehicle treated (2% DMSO in corn oil) and tryptamine (12.5 mg/kg every 48 h) treated chronic progressive EAE mice (n = 20 per group). **(B)** Cumulative score per each mouse of vehicle treated (2% DMSO in corn oil) and tryptamine (12.5 mg/kg every 48 h) treated chronic progressive EAE mice (n = 20 per group). **(C)** Percentage of starting body weight vehicle treated (2% DMSO in corn oil) and tryptamine (12.5 mg/kg every 48 h) treated chronic progressive EAE mice (n = 20 per group).

In order to investigate the immunosuppressive potential of tryptamine, we administered tryptamine (12.5 mg/kg) via i.p injection every 48 h in a murine model of chronic progressive EAE. We analyzed mice daily to observe paralysis symptoms and body weight until any individual mice displayed a severity of symptoms that reflected a moribund state and required euthanasia. At this point, observed at day 13, all mice were humanely euthanized for uniform sample collection. Tryptamine treated mice demonstrated a significant reduction in paralysis symptoms beginning from day 8 that persisted for the remaining duration of the study (Figure 1A). The sum of paralysis scores per mouse further demonstrates a significant reduction in observed paralysis symptoms experienced between tryptamine vs. vehicle treatment groups (Figure 1B). The average weight of mice was also measured throughout the time course of chronic progressive EAE and the data expressed as percent of starting body weight, clearly showed that while the vehicle-treated group started losing weight, especially on days 11–13, the tryptamine-treated mice showed a significant retention of weight when compared to the vehicle controls (Figure 1C). These results together demonstrated that tryptamine treatment of EAE mice ameliorates the clinical symptoms of paralysis and weight loss associated with EAE.

### Tryptamine Treatment Alters Neuroinflammation in EAE Mice

**FIGURE 2:**
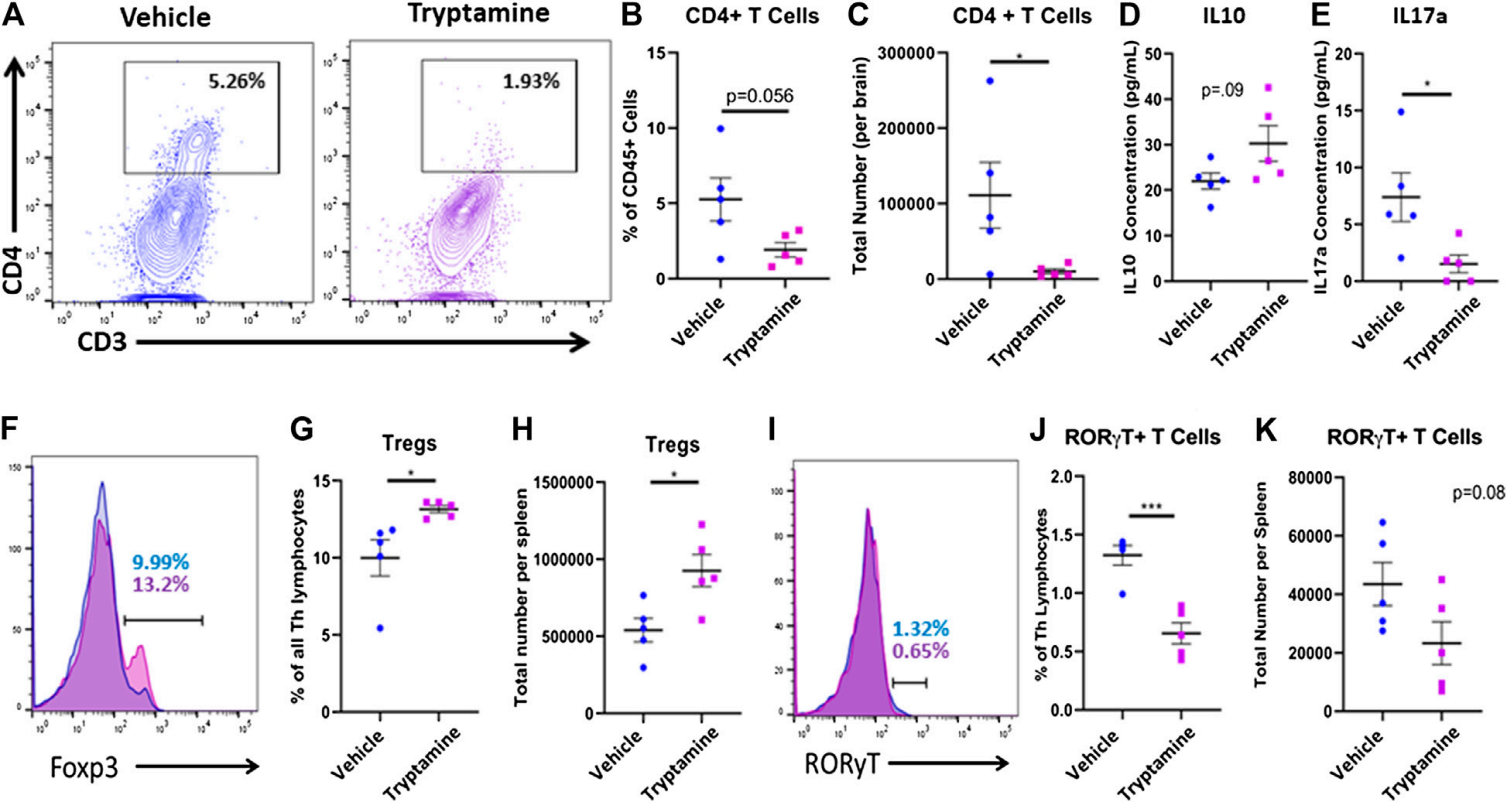
Tryptamine treatment alters neuroinflammation in EAE mice. EAE mice were treated with tryptamine as described in Figure 1 legend and immune response in the CNS and spleens was studied. **(A)** Representative flow plots of CD3 and CD4 expression in CD45^+^ cells in vehicle (blue) and tryptamine-treated (purple) mice. **(B)** CD4^+^ T Cells infiltrating the CNS of EAE mice represented as a percentage of all CD45^+^ cells (n = 5 per group). **(C)** Total number of CD4^+^ T Cells infiltrating the CNS of EAE mice (n = 5 per group). **(D)** Secreted IL10 concentration from cultured mononuclear cells (10 × 10^6^ cells/ml in cRPMI for 24 h isolated from the CNS of vehicle and tryptamine treated mice (n = 5 per group). **(E)** Secreted IL17a concentrations from cultured mononuclear cells (10 × 10^6^ cells/ml in cRPMI for 24 h) isolated from the CNS of vehicle and tryptamine treated mice (n = 5 per group). **(F)** Representative flow plots of Foxp3 expression among CD4^+^ lymphocytes in vehicle (blue) and tryptamine-treated (purple) mice. **(G)** Percentage of Foxp3+ (assumed as Tregs) cells among all CD4^+^ lymphocytes isolated from the spleen of vehicle and tryptamine treated mice (n = 5 per group). **(H)** Total number of Tregs per spleen in vehicle and tryptamine treated mice (n = 5 per group). **(I)** Representative flow plots of RORγT expression among CD4^+^ lymphocytes in vehicle (blue) and tryptamine-treated (purple) mice. **(J)** Percentage of RORγT+ T cells among all CD4^+^ lymphocytes isolated from the spleen of vehicle and tryptamine treated mice (n = 5 per group). **(K)** Total number of RORγT+ T cells per spleen in vehicle and tryptamine treated mice (n = 5 per group).

In order to understand if the attenuation of clinical symptoms by tryptamine results from decreased neuroinflammation, we next investigated the T cell responses in the CNS of these mice. A representative flow plot demonstrated the reduction in CD4^+^ T cells found within the CNS of tryptamine treated mice (Figure 2A). When CNS infiltrating CD4^+^ T cells were analyzed using flow cytometry among the CD45^+^ population (Supplementary Figure S1), we noted that while the percentage of CD4^+^ T cells did not significantly decrease in comparison to the vehicle group (Figure 2B), there was significant reduction in the total number of infiltrating CD4^+^ T cells present in the CNS (Figure 2C). Analysis of anti-inflammatory (IL-10) and pro-inflammatory (IL-17) cytokines secreted by cultured mononuclear cells isolated from the CNS revealed that tryptamine treated mice displayed no significant change in IL-10 secretion (Figure 2D) while significantly suppressing IL-17 secretion in comparison to the vehicle group (Figure 2E). Because the MOG antigen is injected peripherally, we also tested the spleens of the tryptamine for Tregs vs. Th-17 cells to test if tryptamine also altered the immune response in the periphery. CD4^+^ T Cells were identified from the spleens as being CD3^+^CD4^+^CD8^−^ prior to looking at transcription factor expression (Supplementary Figure S2). This increase in the Treg population of tryptamine treated mice is represented with overlaying histograms demonstrating Foxp3 expression among CD4^+^ T cells between the groups (Figure 2F). We found that tryptamine treated mice displayed a higher proportion (Figure 2G) as well as total number (Figure 2H) of anti-inflammatory Tregs in comparison to the spleens of vehicle treated mice. After discovering an increase in the anti-inflammatory Treg subset, we then studied the pro-inflammatory counterpart of RORγT+ T cells. The spleens of tryptamine treated mice demonstrated a reduction in the percentage of RORγT+ T cells as shown by a representative histogram (Figure 2I). Tryptamine treatment significantly reduced the proportion of the pro-inflammatory RORγT T cell subset within the spleens in comparison to the vehicle group (Figure 2J) while displaying a trend in the total number of Th17 lymphocytes, however this trend was not statistically significant (Figure 2K). Collectively these results showed that treatment with tryptamine suppresses inflammation in both the periphery and in the CNS.

### Tryptamine Inhibits the Inflammatory Capacity of MOG35-55 Specific CD4^+^ T Cells and Ameliorates Their Encephalitogenic Activity in an Aryl Hydrocarbon Receptor Dependent Manner

In order to better demonstrate that tryptamine ameliorates EAE by regulating CD4^+^ T cell reactivity in an AHR-dependent manner we tested the immunosuppressive capacity of tryptamine on MOG35-55 reactive T cells donated from wild WT and Lck-Cre AHR^flox/flox^ mice prior to the induction of CD4^+^ T cell mediated passive polyclonal EAE. Briefly, C57BL/6 wild-type and Lck-Cre AHR^flox/flox^ mice were immunized with MOG35-55 antigen and immune cells from the spleens and lymph nodes were further activated with a combination of MOG35-55 and pro-inflammatory cytokines IL-2, IL-12 and IL-18 *in vitro*, in the presence or absence of tryptamine (100 µM). Following a 72 h incubation the cell suspensions were then transferred into naïve wild-type mice via retroorbital injection. The development of EAE symptoms were studied on a daily basis as described in *Materials and Methods*.

**FIGURE 3:**
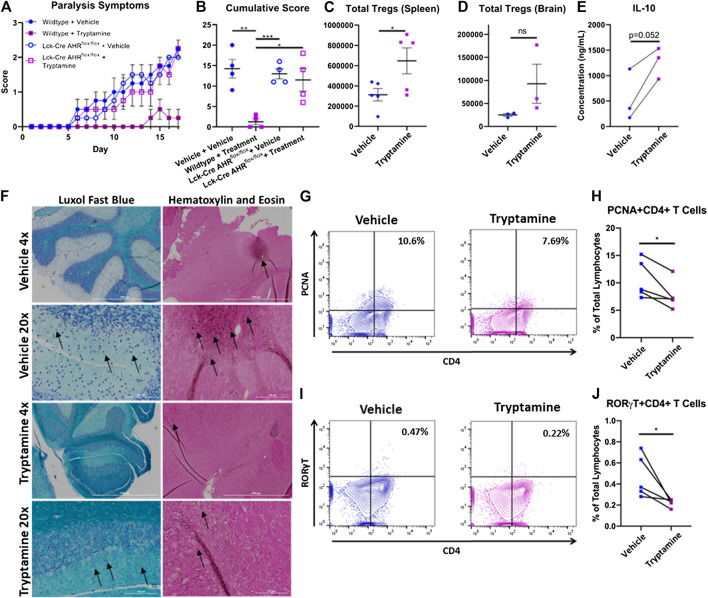
Tryptamine inhibits the inflammatory capacity of MOG specific CD4^+^ T Cells and ameliorates their encephalitogenic activity in an aryl hydrocarbon receptor dependent manner. **(A)** Paralysis symptoms of chronic progressive polyclonal passive EAE mice. Mice received a retro orbital injection of 4 million live cells that were cultured under stimulating conditions that promote the expansion of encephalitogenic CD4^+^ T cells. Donor cells were derived from WT and Lck-Cre AHR^flox/flox^ mice and treated *ex-vivo* in the presence of vehicle (DMSO) or 100 µM tryptamine prior to induction of disease and divided into four groups (wildtype + vehicle, wildtype + tryptamine treatment, Lck-Cre AHR^flox/flox^ + vehicle, and Lck-Cre AHR^flox/flox^ + tryptamine treatment; n = 4 per group). **(B)** Cumulative score per each passive polyclonal EAE group (n = 4 per group). **(C)** Total number of Tregs per brain from chronic progressive polyclonal passive EAE mice given cells treated *ex vivo* with either vehicle or tryptamine (n = 5). **(D)** Total number of Tregs per spleen from chronic progressive polyclonal passive EAE mice given cells treated with either vehicle or tryptamine (n = 3). **(E)** Production of IL-10 among mononuclear cells isolated from MOG immunized mice and stimulated with 20 µg/ml MOG35-55 *in vitro* in the presence or absence of 100 µM tryptamine (10 × 10^6^ cells/ml in cRPMI with for 24 h, n = 3). **(F)** Luxol Fast Blue and Hematoxylin and Eosin staining of the CNS from chronic progressive polyclonal EAE mice that received either tryptamine or vehicle treated encephalitogenic T Cells. Black arrows indicate neuronal bundles (Luxol Fast Blue) and areas with evidence of cellular infiltration (Hematoxylin and Eosin). **(G)** Representative flow plots of PCNA and CD4 expression among mononuclear cells isolated from MOG immunized mice and stimulated with MOG35-55 *in vitro* in the presence or absence of tryptamine. **(H)** Percentage of PCNA+CD4^+^ lymphocytes among all lymphocytes in mononuclear cells isolated from MOG immunized mice and stimulated with 20 µg/ml MOG35-55 *in vitro* in the presence or absence of 100 µM tryptamine (10 × 10^6^ cells/ml in cRPMI with for 24 h, n = 5). **(I)** Representative flow plots of RORγT and CD4 expression among mononuclear cells isolated from MOG immunized mice and stimulated with MOG35-55 *in vitro* in the presence or absence of tryptamine. **(J)** Percentage of RORγT+CD4^+^ lymphocytes among all lymphocytes in mononuclear cells isolated from MOG immunized mice and stimulated with 20 µg/ml MOG35-55 *in vitro* in the presence or absence of 100 µM tryptamine (10 × 10^6^ cells/ml in cRPMI with for 24 h, n = 5).

The mice which received transfer of cells from wildtype+Vehicle, Lck-Cre AHR^flox/flox^ +Vehicle and Lck-Cre AHR^flox/flox^ +Tryptamine demonstrated significant paralysis symptoms, while those that received transfer of cells from wildtype + tryptamine group developed only mild paralysis symptoms (Figure 3A). These results are further corroborated by the cumulative scores of paralysis scores per mouse (Figure 3B). Flow cytometry analysis of the spleen (Figure 3C) and CNS (Figure 3D) revealed that mice which received a transfer of tryptamine treated encephalitogenic T cells from wildtype mice displayed increased Treg abundance when compared to similar cells that were treated with vehicle. Measurement of IL-10 in *in vivo* sensitized cells with MOG that were activated *in vitro* with MOG in the presence of tryptamine showed an increase in IL-10 secretion when compared to vehicle controls, but it was not statistically significant (Figure 3E). Histological analysis of brain in passive EAE experiments showed a retention of myelin indicated by a deeper intensity of Luxol fast blue staining in the areas surrounding the dark blue stained neuronal cell bodies and a reduction of immune cell infiltration denoted by a lack of dark colored hematoxylin stained infiltrates in the mice that received cells treated with tryptamine *in vitro* when compared to mice that received similar cells treated with vehicle (Figure 3F).

Because culture of MOG-sensitized T cells *in vitro* with tryptamine and adoptive transfer led to decreased EAE when compared to vehicle-treated cells, we next investigated the mechanisms involved. Representative flow plots demonstrated the percentage of CD4^+^ and PCNA+ cells among all lymphocytes collected from immunized mice stimulated *in vitro* with MOG35-55 in the presence or absence of tryptamine demonstrated a reduction in proliferative activity (Figure 3G). Tryptamine treatment *in vitro* reduced the percentage of proliferating CD4^+^ T cells as demonstrated by PCNA+ cells (Figure 3H). Also, tryptamine treatment *in vitro* significantly reduced the percentage of CD4^+^ RORγT as shown in a representative flow cytometric analysis (Figure 3I) and the percentage of such cells from multiple samples (Figure 3J).

### Tryptamine Treatment in EAE Promotes Butyrate Production by the Gut Microbiota

**FIGURE 4:**
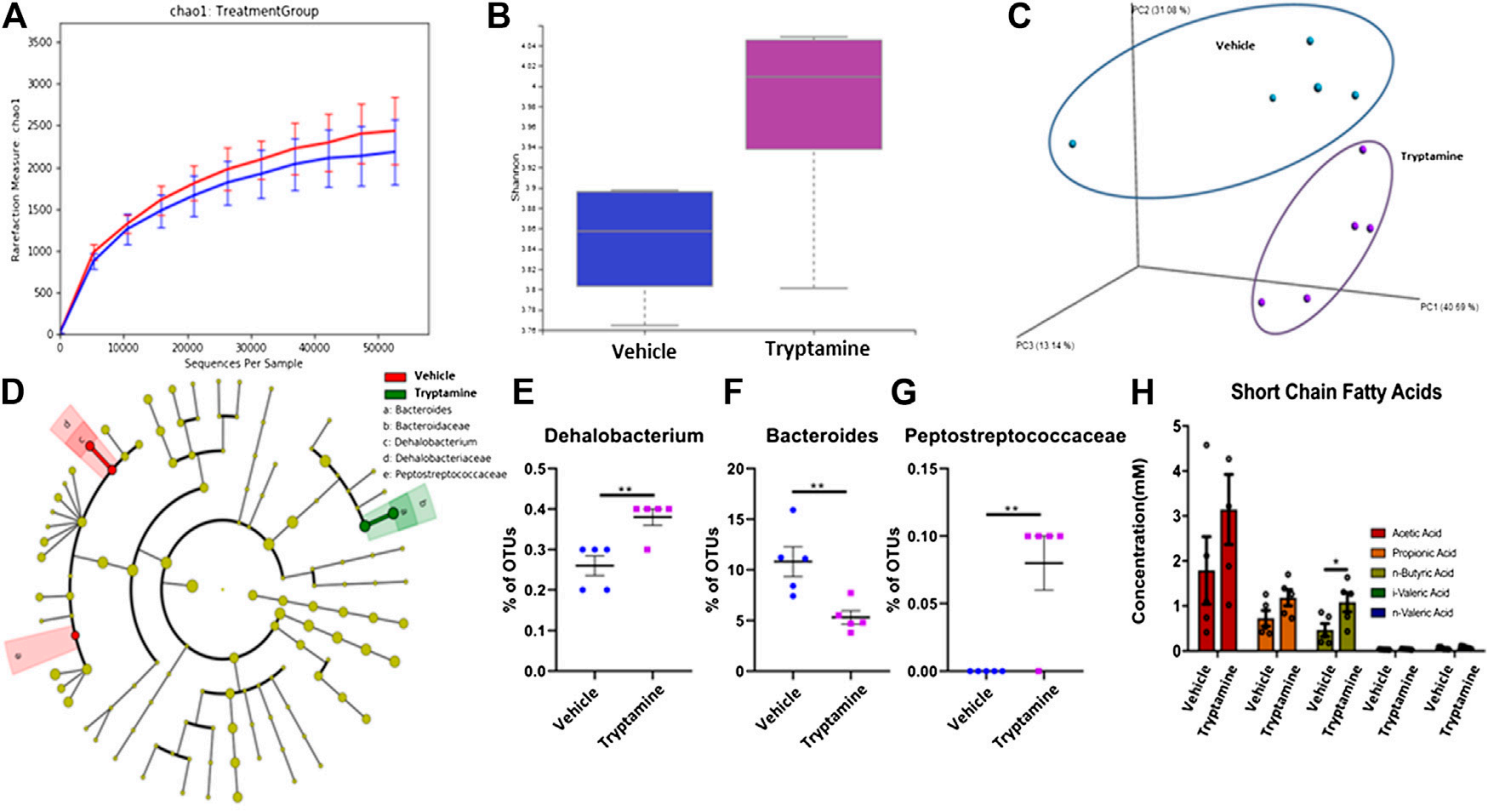
Tryptamine treatment in EAE promotes butyrate production by the gut microbiota. **(A)** Sequenced reads were analyzed with Nephele software to plot the chao index indicating average number of unique 16s reads per sample of cecal content between vehicle and tryptamine treated mice (n = 5 per group). **(B)** Sequenced reads were analyzed with Nephele software to plot the shannon index between samples of cecal content between vehicle and tryptamine treated mice (n = 5 per group). **(C)** PCoA plot displaying unique clustering of the cecal microbiota among mice treated with vehicle vs. mice treated with tryptamine (n = 5 per group). **(D)** LEfSe analysis indicating the observed changes within the cecal microbiota content. **(E)** Relative abundance of Dehalobacterium within the cecal content based on 16s sequencing reads (n = 5 per group). **(F)** Relative abundance of *Bacteroides* within the cecal content based on 16s sequencing reads (n = 5 per group). **(G)** Relative abundance of Peptostreptococcaceae within the cecal content based on 16s sequencing reads (n = 5 per group). **(H)** SCFA concentration within the cecal content of vehicle vs. tryptamine treaded mice (n = 5 per group).

Previous literature has demonstrated that AHR ligands exert their anti-inflammatory properties by a combination of direct interactions on the immune system and indirect interactions mediated by the GI microbiota (Lefever et al., 2016; Brawner et al., 2019; Busbee et al., 2020). For this purpose, we studied the effects of tryptamine treatment *in vivo* on the composition of the cecal microbiota in EAE mice. The chao1 index (Figure 4A) and Shannon index (Figure 4B) of 16s sequencing reads indicated there were slight increases in the alpha diversity of the cecal microbiome composition in tryptamine treated mice. PCoA plotting demonstrated that the cecal microbiota of treated mice was distinctly clustered from the vehicle group (Figure 4C). LEfSe analysis was conducted on the 16s sequencing outputs to distinguish differentially regulated bacterial phylogenies (Figure 4D). Three LEfSe identified phylogenies in *Dehalobacterium* (Figure 4E), *Bacteroides* (Figure 4F) and *Peptostreptococcaceae* (Figure 4G) to be significantly altered in tryptamine-treated groups when compared to the vehicle controls. When Short Chain Fatty Acids (SCFAs) were quantified using mass spec the data revealed a significant increase in n-Butyric Acid following tryptamine treatment when compared to vehicle controls (Figure 4H). These results suggested that tryptamine treatment alters the microbiota in the gut and promotes the induction of n-Butyric Acid.

## Discussion

MS is an incurable autoimmune disorder that is perpetuated by a complex etiology contributing to excessive inflammation occurring within the CNS. Previous studies have established that the gut microbiota shapes the host immunity in neuroinflammatory disorders and, when there is dysbiosis, it can trigger autoimmune encephalomyelitis (Jangi et al., 2016; Berer et al., 2017; Cekanaviciute et al., 2017; Lee and Kim, 2017; Dopkins et al., 2018). Due to the critical role played by the microbiota in the etiology of inflammatory disorders, we studied the immunomodulatory potential of tryptamine, a microbial metabolite that is produced in large quantities within the GI tract. Tryptamine production within the gut has been characterized within two known commensal microorganisms that inhabit the GI tract of homeothermic organisms, *Clostridium sporogenes* and *Ruminococcus gnavus* (Williams et al., 2014). Clinical studies have demonstrated that *Clostridium* species are decreased in MS patients in comparison to healthy controls, while there are conflicting reports that demonstrate primarily increased abundance of Ruminococcus species in MS patients with one specific cohort of adolescent MS patients showing decreased Ruminococcaceae abundance (Shahi et al., 2017; Chu et al., 2018). These studies did not identify the bacterial composition of *Ruminococcus gnavus* or *Clostridium sporogenes* at the species level and do not definitively state that these tryptamine producing species specifically are regulated in MS patients. However, these studies in combination with our work strengthen the suggestion that hindered tryptamine production within the GI tract may affect the clinical severity of MS due to the upper level phylogenies of these bacterial species being significantly regulated in MS patients when compared to healthy controls. Further studies are clearly needed to determine if tryptamine deficiency is associated with susceptibility to MS. This may involve measuring the concentrations of tryptamine within the intestinal tracts of patients, as well as quantifying the relative abundance of tryptamine producing bacterial species and the tryptophan decarboxylase enzyme.

Previous studies have demonstrated the strong association between exogenous AHR ligands regulating autoimmune neuroinflammation in an AHR-dependent manner (Rouse et al., 2013; Rouse et al., 2014; Ginwala et al., 2016). Our study demonstrates that tryptamine possesses a potent neuroprotective role in the context of EAE by limiting excessive neuroinflammation and ameliorating the associated paralysis symptoms by directly inhibiting encephalitogenic T cell activity in an AHR-dependent manner. With these data we suggest that interactions between host immunity and exogenously produced tryptamine exerts significant suppression of neuroinflammation by preventing and inhibiting encephalitogenic T cell activity. We confirmed this by showing that transfer of WT encephalitogenic T cells treated with tryptamine *ex-vivo* into wild-type mice caused the induction of very mild form of EAE and significant attenuation of neuroinflammation when compared to similar cells treated with the vehicle which induced robust EAE and inflammation in the CNS. Interestingly, transfer of encephalitogenic T cells lacking a functional AHR, treated with tryptamine *ex-vivo*, displayed no reduction in encephalitogenic capacity in comparison to control groups. These data confirmed that tryptamine acts through AHR. Mechanistically, we found that treatment of encephalitogenic cells with tryptamine *in vitro*, inhibited the proliferation of CD4^+^ T cells and inhibited RORγ T cells, and furthermore, when such cells were transferred into wild-type mice, they induced more Tregs than similar cells treated with the vehicle. The fact that encephalitogenic T cells lacking a functional AHR when treated with the vehicle *ex-vivo*, were able to induce the same levels of EAE as wild-type T cells treated with the vehicle also suggested that AHR expression on T cells per se may not play a role in the induction of EAE in the adoptive transfer model of EAE tested in the current investigation. Confirmation that the immunosuppressive effects of tryptamine are dependent on AHR expression coincides with previous literature demonstrating that a variety of AHR ligands promote an immunotolerant phenotype in T cell subsets (Ye et al., 2017).

Previous literature has demonstrated that a primary mechanism by which AHR ligands limit excessive immune responses in autoimmune disorders is by regulating a dysbiotic microbiota and promoting the production of the SCFAs (Ji and Qu, 2019; Busbee et al., 2020). Additionally, it has been suggested by previous studies that microbially produced tryptamine affects intestinal motility and the enteric nervous system in a manner that may have effects regulating the GI flora (Williams et al., 2014; Wlodarska et al., 2015; Gao et al., 2018). For these purposes, we investigated the changes observed within the cecal contents of EAE mice that received tryptamine treatment in comparison with vehicle controls by use of 16s sequencing and mass spectrometry. Within the cecal contents of tryptamine treated mice, there was a significant increase in the abundance of *Peptostreptococcaceae*, a family of Gram-positive bacteria which was shown to be downregulated in patients suffering from the autoimmune demyelinating disease, Neuromyelitis Optica (Shi et al., 2020). While any potential mechanisms with which *Peptostreptococcaceae* species directly limit autoimmune neuroinflammation are not currently defined, *Peptostreptococcaceae* is normal constituent of a healthy gastrointestinal microbiota that contributes to maintaining intestinal homeostasis and has been shown to be significantly depleted under dysbiotic conditions (Leng et al., 2016; Fan et al., 2017). Mass spectrometry analysis revealed that the tryptamine treatment significantly increased the concentration of the anti-inflammatory bacterial metabolite butyrate within the cecal contents of EAE mice. Cecal butyrate content has been established to induce the immunosuppressive Treg lymphocytes, which have been correlated with a positive outcome in autoimmune disorders and EAE (Furusawa et al., 2013; Mizuno et al., 2017). Here we suggest that tryptamine treatment in inflammatory disorders promotes an anti-inflammatory metabolomic profile within the gut through promoting the production of butyrate. The translational impact of tryptamine enacting on the immune system, enteric nervous system and intestinal epithelium to further promote an anti-inflammatory gut microbiota is pivotal due to tryptamine’s potential role as a novel messenger enabling crosstalk between the microbiota and host immunity at barrier sites. This promotion of butyrate suggests that the production of tryptamine within a healthy gastric flora may play an important role in further influencing the immune system to maintain a healthy gastric flora and prevent excessive inflammation. Therefore, normal tryptamine production may be a negative regulator of inflammation involved in maintaining homeostatic conditions between the GI microbiota and host immunity by promoting Tregs, and when absent, leaves inflammation and bacterial dysbiosis unregulated as is observed in inflammatory disorders (André et al., 2019; Leng et al., 2016; Furusawa et al., 2013).

In summary, this study demonstrates for the first time that administration of tryptamine attenuates a murine model of MS by suppressing neuroinflammation and adds to the growing dataset demonstrating that microbial metabolism within the GI tract plays an important role in regulating neuroinflammation through the production of AHR ligands. Our study has demonstrated that tryptamine under inflammatory conditions shifts the T cell landscape toward an immunosuppressive state and directly interacts with myelin-reactive T cells to inhibit neuroinflammation in an AHR dependent manner. In combination with directly suppressing the immune response, tryptamine results in anti-inflammatory shifts in the metabolome of the GI microbiota suggesting that tryptamine may play an important role in the crosstalk between host and flora required to maintain intestinal homeostasis. In the long term, characterizing the immunomodulatory capacity of tryptamine and other AHR ligands produced by normal flora will help further define the complex etiology of autoimmune disorders and potentially translate into clinical applications such microbiome-based therapies.

## Data Availability Statement

The datasets presented in this study can be found in online repositories. The names of the repository/repositories and accession number(s) can be found below: https://www.ncbi.nlm.nih.gov/, SAMN16476358, SAMN16476359, SAMN16476360, SAMN16476361, SAMN16476362, SAMN16476363, SAMN16476364, SAMN16476365, SAMN16476366, SAMN16476367.

## Ethics Statement

The animal study was reviewed and approved by University of South Carolina Institutional Animal Care and Use Committee.

## Author Contributions

ND, PN, and MN conceived the ideas and hypothesis presented throughout the manuscript. Sample collection was performed by ND with experimentation on collected samples conducted by ND, WB, KM, and MW. ND wrote the manuscript and developed the accompanying figures with support from PN and MN.

## Funding

Supported by NIH grants P01AT003961, P20GM103641, R01ES030144, R01AI129788, and R01AI123947.

## Conflict of Interest

The authors declare that the research was conducted in the absence of any commercial or financial relationships that could be construed as a potential conflict of interest.
