# Supplementary material for: Tryptamine Attenuates Experimental Multiple Sclerosis Through Activation of Aryl Hydrocarbon Receptor
Source: Front Pharmacol. 2021 Jan 25;11:619265. doi: 10.3389/fphar.2020.619265 (PMC7868334; doi:10.3389/fphar.2020.619265)
Supplement: Supplementary file 1 [file presentation1.pptx]

## Slide 1
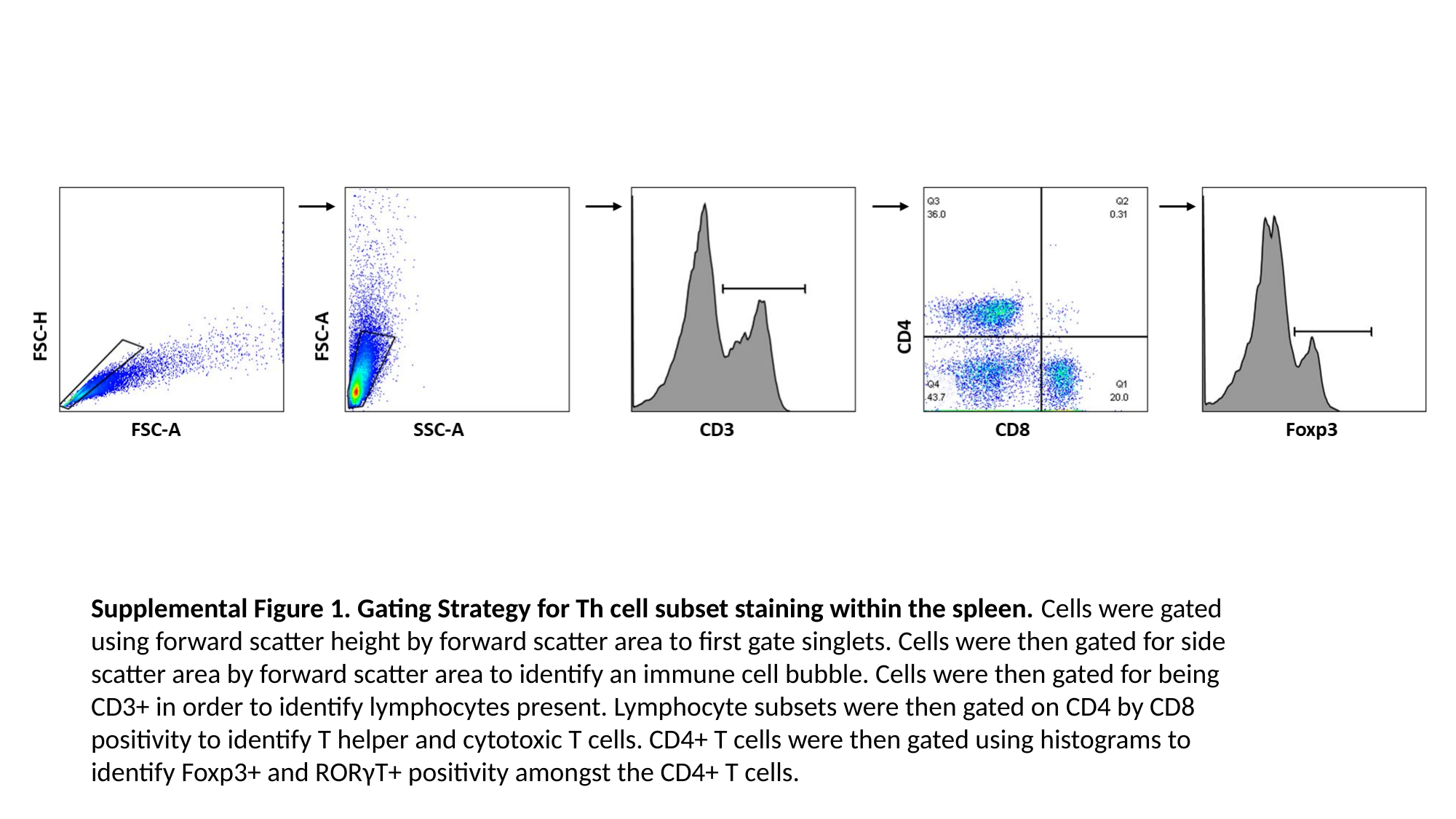

Supplemental Figure 1. Gating Strategy for Th cell subset staining within the spleen. Cells were gated using forward scatter height by forward scatter area to first gate singlets. Cells were then gated for side scatter area by forward scatter area to identify an immune cell bubble. Cells were then gated for being CD3+ in order to identify lymphocytes present. Lymphocyte subsets were then gated on CD4 by CD8 positivity to identify T helper and cytotoxic T cells. CD4+ T cells were then gated using histograms to identify Foxp3+ and RORγT+ positivity amongst the CD4+ T cells.

## Slide 2
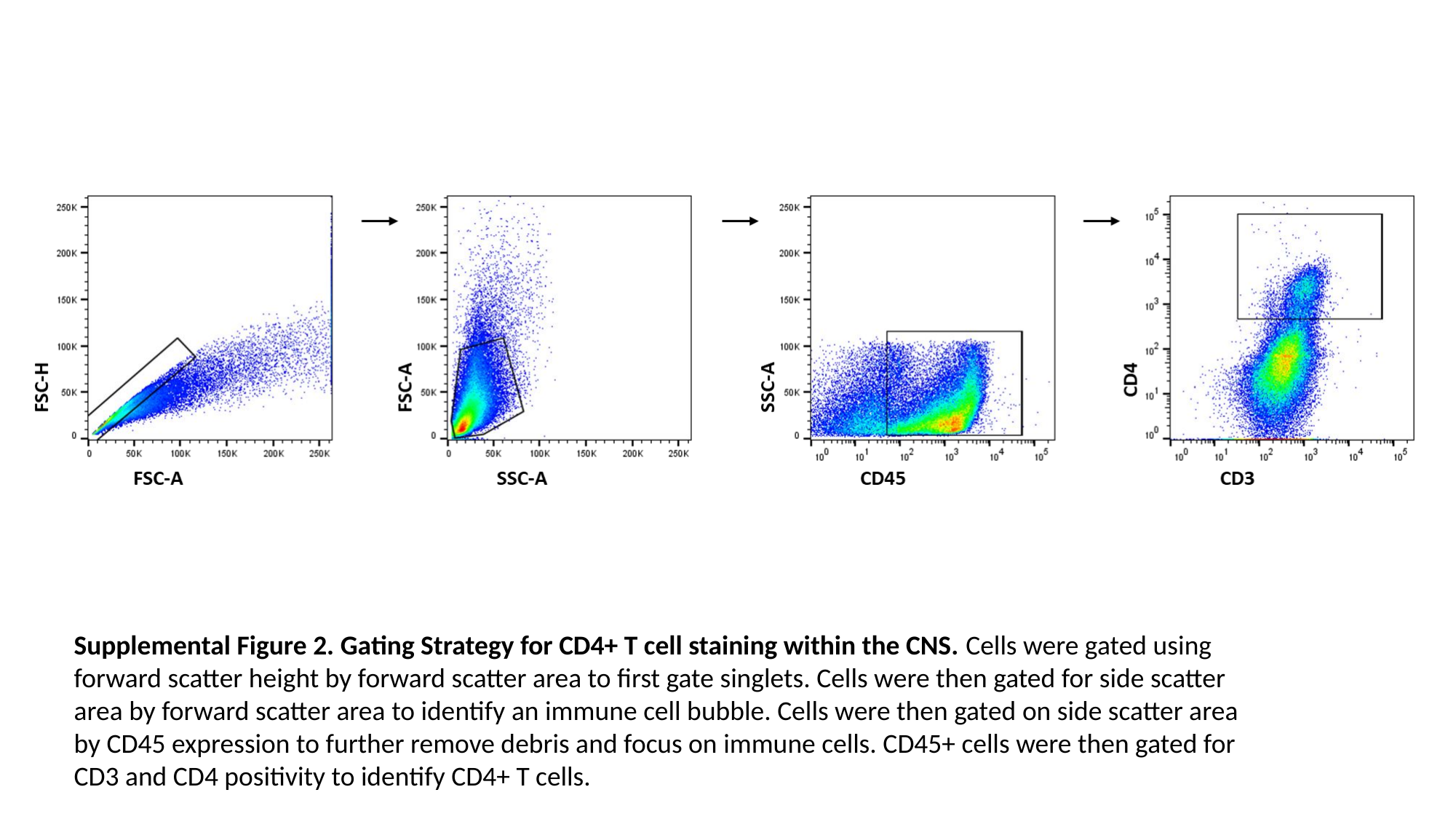

Supplemental Figure 2. Gating Strategy for CD4+ T cell staining within the CNS. Cells were gated using forward scatter height by forward scatter area to first gate singlets. Cells were then gated for side scatter area by forward scatter area to identify an immune cell bubble. Cells were then gated on side scatter area by CD45 expression to further remove debris and focus on immune cells. CD45+ cells were then gated for CD3 and CD4 positivity to identify CD4+ T cells.
